# A high-accuracy lightweight network model for X-ray image diagnosis: A case study of COVID detection

**DOI:** 10.1371/journal.pone.0303049

**Published:** 2024-06-18

**Authors:** Shujuan Wang, Jialin Ren, Xiaoli Guo

**Affiliations:** College of Mathematics and Information Science, Zhengzhou University of Light Industry, Zhengzhou, China; New York University Abu Dhabi, UNITED ARAB EMIRATES

## Abstract

The Coronavirus Disease 2019(COVID-19) has caused widespread and significant harm globally. In order to address the urgent demand for a rapid and reliable diagnostic approach to mitigate transmission, the application of deep learning stands as a viable solution. The impracticality of many existing models is attributed to excessively large parameters, significantly limiting their utility. Additionally, the classification accuracy of the model with few parameters falls short of desirable levels. Motivated by this observation, the present study employs the lightweight network MobileNetV3 as the underlying architecture. This paper incorporates the dense block to capture intricate spatial information in images, as well as the transition layer designed to reduce the size and channel number of the feature map. Furthermore, this paper employs label smoothing loss to address the inter-class similarity effects and uses class weighting to tackle the problem of data imbalance. Additionally, this study applies the pruning technique to eliminate unnecessary structures and further reduce the number of parameters. As a result, this improved model achieves an impressive 98.71% accuracy on an openly accessible database, while utilizing only 5.94 million parameters. Compared to the previous method, this maximum improvement reaches 5.41%. Moreover, this research successfully reduces the parameter count by up to 24 times, showcasing the efficacy of our approach. This demonstrates the significant benefits in regions with limited availability of medical resources.

## 1 Introduction

In recent years, a widespread COVID-19 crisis has erupted globally, causing massive infections and deaths worldwide. The impact has extended across various domains, including the economy, society, and mental health. The continued diffusion of the COVID-19 pandemic has hindered global connectivity [1]. Currently, although the promotion of vaccines and preventive measures has to some extent slowed down the spread of the virus, there still exists the risk of new variants emerging and the outbreak of new epidemics. To effectively combat the spread of COVID-19 in society, accurate diagnosis as early as possible plays a crucial role. The widely used testing method currently employed is Reverse Transcription Polymerase Chain Reaction (RT-PCR), which involves the test and amplification of viral RNA extracted from nasopharyngeal swabs or sputum samples. However, RT-PCR has significant limitations. Firstly, the testing process is time-consuming and usually takes several hours or more. Such delays can have implications for outbreak control and the timely implementation of necessary measures. Additionally, the accessibility of RT-PCR testing kits is limited, particularly in developing countries [2, 3]. These restrictions are risky for people with COVID-19. Therefore, medical imaging tests are performed first to initially detect COVID-19, followed by RT-PCR tests to help doctors make an accurate final diagnosis [4]. Computed Tomography (CT) and Chest X-ray (CXR) are two common medical imaging techniques used for COVID-19 detection, each with unique advantages [5].

CT images excel in their superior penetration, providing high-resolution anatomical structures, and generating three-dimensional information to support stereoscopic anatomy. In diagnosing complex conditions such as deep-seated tumors, brain diseases, and abdominal issues, CT typically offers more accurate information, aiding clinicians in critical diagnostic and therapeutic decision-making. However, it is important to note that CT scans, compared to CXRs, require a higher radiation dosage, potentially increasing the risk of radiation exposure to patients. And, for patients, CT images are far more expensive than CXR images [4]. In contrast, Chest X-ray (CXR) exhibits advantages such as low radiation exposure, rapid acquisition, and cost-effectiveness. CXR is well-suited for screening and tracking pulmonary diseases, especially in emergency situations where its capability to swiftly capture images aids in the prompt assessment of lung issues like pneumonia, tuberculosis, and lung cancer. Furthermore, due to its relatively lower cost, CXR finds widespread application in regions with limited medical resources. In certain scenarios, such as screening for pneumonia, pulmonary edema, or tuberculosis, CXR often proves to be the more appropriate choice [6]. However, manually examining a large number of CXR images to distinguish COVID-19 patients from others is a difficult and time-consuming task [7]. Therefore, developing an automated technique for accurate COVID-19 diagnosis is necessary and Deep Learning(DL) methods have been suggested to solve this challenge [8].

Convolutional Neural Network (CNN), a subdomain of DL algorithms, has been widely studied and proven effective in the detection of COVID-19 [8]. Such as, the researchers utilized several pre-trained models to detect three categories, including COVID-19, Viral Pneumonia, and Normal cases by CXR images. The researchers indicated that Visual Geometry Group 19(VGG19) achieved the highest accuracy at 93.48% [9]. However, these models remain challenging in the field of COVID-19 because of the following two problems. In the early stages of the COVID-19 pandemic, researchers faced a huge difficulty due to the lack of reliable data. And now larger datasets have become accessible, enabling more recent studies to leverage these extensive datasets to ensure the accuracy of their models [10]. The current model training processes are conducted on a powerful Graphics Processing Unit (GPU), bringing about a notable quantity of parameters. It is hard to practice especially for the people in regions with limited medical resources. All the problems need the development of models that are more suitable for real-world deployment [11].

To address the previously mentioned issues, this paper proposes an improved lightweight network called Dense MobileNetV3. This model enhances the ability to capture complex image information at different layers by incorporating the Dense Block into the lightweight MobileNetV3 architecture. Firstly, the model is trained using transfer learning and subsequently fine-tuned to optimize its performance. Secondly, a pruning operation is employed to decrease the parameters of the improved method. Thirdly, to evaluate the capabilities of the improved model, accuracy, sensitivity, specificity, and precision index are assessed by using an open accessible chest X-ray image database. Finally, this experiment attains an impressive accuracy of 98.71% with a parameter count of 5.94 million, which is up to 5.41% more accurate compared to the previous methods and successfully reduces the parameter count by up to 24 times. The reduction in model parameters leads to a significant decrease in computational resource utilization and memory requirements. The outcomes demonstrate its competitiveness when compared to heavyweight models, highlighting its practicality. Furthermore, the enhanced approach surpasses certain currently available lightweight networks in terms of accuracy, underscoring its efficacy. This research aims to address the challenge of achieving efficient COVID-19 detection on devices with limited computational resources, a matter of particular significance for remote areas and situations with scarce medical resources.

The primary highlights of this study are summarized and expounded upon as follows:

- A lightweight network Dense MobileNetV3 is developed for the early identification of individuals with COVID-19, Viral Pneumonia, or Normal cases using chest X-ray images. It achieves an impressive accuracy of 98.71% with just 5.94 million parameters.
- This paper combines the Dense Blocks to extract and concatenate image features at various scales in the spatial dimension, resulting in high-level attributes. Additionally, this study mitigates the inter-class similarity effect by employing label smoothing loss.
- Building upon the highly acclaimed lig htweight network MobileNet and achieving improvements by incorporating pruning techniques, led to a significant reduction in the parameter count.

The subsequent parts of the article are structured as the following: Section 2 presents recent methodologies. Section 3 provides a detailed description of the improved method mentioned above. Section 4 provides details about the dataset and training. Section 5 presents the experimental results and discussion, and Section 6 concludes with a summary of the article.

## 2 Related work

Since 2012, deep neural network based on CNN has made significant advancements and achieved impressive results in the ImageNet competition [12]. And the researchers have increasingly directed their attention towards leveraging machine learning techniques for medical image analysis. Among the diverse machine learning methods suggested in the relevant literature, CNN has demonstrated remarkable efficacy in various applications relevant to COVID-19 prediction and diagnosis. Specifically, CNN has been utilized for expeditious and precise diagnosis of COVID-19 infection by CXR images [13].

Ahamed et al. [14] proposed a modified ResNet50V2 architecture is proposed as the detection model. The model was trained using a dataset consisting of chest CT scans and X-ray images. Aggregated data sets are preprocessed through sharpening filters before entering them into the proposed model. Using CXR images, the model achieved 97.242% accuracy on the three-classification task (COVID-19/Normal/Bacterial pneumonia) and 98.954% accuracy on the two-classification task (COVID-19/Viral pneumonia). The model used chest CT scan images to obtain a combined accuracy of 99.012% for three types of cases (COVID-19/normal/community-acquired pneumonia) and 99.99% for two types of cases (normal/COVID-19). Such high accuracy allows radiologists to identify and rapidly diagnose COVID-19 using basic but widely available equipment. It provides valuable reference for the follow-up research.

Gupta et al. [15] developed an effective computer-aided technique to diagnose COVID-19 individuals. The researchers performed fine-tuning on pre-trained deep learning to capture features. These features were combined by employing a specific integrated stacking method, resulting in a novel approach named InstaCovNet-19. The experiment findings demonstrated that the model achieved 99.08% accuracy in the three-classification task and 99.53% accuracy in the binary-classification task. Additionally, the article mentioned that Inception-V3 achieved an accuracy of 97.00% with 24 million parameters for the three-classification task.

Wang et al. [16] designed a method named COVID-Net, which was among the pioneering open-source network architectures developed for detecting COVID-19 by using chest X-ray images. The authors used interpretative methods to understand how COVID-19 was predicted and to identify the key factors associated with COVID-19 cases. This approach aimed to assist doctors in performing better screening and review the decision-making process for COVID-19 in a responsible and transparent manner. COVID-Net has an accuracy of 93.3% in the three-classification task with approximately 11.75 million parameters.

Ukwandu et al. [17] developed three lightweight architectures by fine-tuning the MobileNetV2 algorithm for diagnosing COVID-19 patients by using CXR images. These models were introduced for three classification and two classification tasks. The accuracy for the three classification tasks reached 94.5%, and the total number of parameters was 3.53 million. The results demonstrated comparable capability to current methods while greatly boosting the efficiency of implementation.

Hussain et al. [18] introduced CoroDet to detect COVID-19 by using CXR and CT images. This method reached high accuracy in categorizing COVID-19 cases into different severity levels. The authors also presented the largest dataset prepared for evaluating classification algorithms, which was crucial for the development and validation of such models. However because of the limitations of the low computing power of hardware facilities, the researchers used a small amount of data to train the model. As a result, the CoroDet obtained an accuracy of 94.2% for three classification tasks.

Zebin and Rezvy [19] categorized COVID-19 from public datasets by using the transfer learning algorithm. They applied multiple pre-trained convolutional structures to capture features and achieved a classification accuracy of 90% with VGG16, 94.3% with ResNet50, and 96.8% with EfficientNetB0, respectively. EfficientNetB0 achieved the best result with 5.3 million parameters.

Sahoo et al. [20] presented a multi-stage computer-aided framework for classifying normal and COVID-19 cases in chest X-rays (CXRs). The work addresses irrelevant features from non-lung areas through custom layer fine-tuning. Moreover, it incorporates an infection segmentation module using fuzzy rank ensemble methods, thereby enhancing model interpretability. Experimental results demonstrate the effectiveness of this segmentation-based classifier, achieving an accuracy of 98.05%, precision of 97.58%, and sensitivity of 97.96%.

Ghassemi et al. [21] proposed a method built upon pre-trained deep neural networks, which incorporates Cycle-Generative Adversarial Networks (CycleGAN) for effective data augmentation on CT image datasets, thereby achieving an impressive accuracy rate of 99.60%. However, it is noteworthy that while this approach delivers remarkable precision, its practical application inevitably leads to increased costs associated with CT scans, including both diagnostic expenses and patient radiation exposure.

In this field of COVID-19 classification, existing CNN methods have obtained some encouraging achievements. However, there are still some drawbacks. Firstly, many models face challenges due to limited datasets, which makes adequate training difficult. Secondly, the heavyweight models require more computing resources and storage space, limiting their application in resource-constrained regions. Thirdly, a large number of model parameters can lead to overfitting, especially with small datasets. Until now, the availability of samples increased through public repositories like GitHub and Kaggle. Then researchers aim to find a better equilibrium between model capability and the count of parameters. This paper proposes the Dense MobileNetV3, an improved version of the lightweight network MobileNetV3. With the dense block, this paper achieves a higher accuracy with minimal parameters than existing models.

## 3 Approach

This article proposes a novel model called Dense MobileNetV3 based on MobileNetV3-large. In this improved model, the Dense Block is added behind the SE (Squeeze-and-Excitation) structure in the second half of the original network structure. This improvement aims at enhancing the model’s ability to discern subtle differences by reinforcing feature reuse and multi-level feature integration, thereby increasing classification accuracy. The transition layer is immediately placed following the Dense Block structure, serving to downsample and decrease both the size and the number of channels in the feature map. Table 1 describes the whole framework of the model presented above. In order to optimize the training process, this study combines cross entropy loss and label smoothing techniques. This combination minimizes the similarity effect between different classes, enhancing the model’s ability to differentiate diverse cases. And the transfer learning strategy is adopted to train the model, making full use of the weight of the pre-trained MobileNetV3 model, which significantly speeds up the model convergence and optimizes the initialization performance. Simultaneously, the channel pruning technique was leveraged during the training process to automatically identify and prune insignificant channel parameters, thereby realizing a targeted slimming down of the model. This procedure aims at removing redundant parameters while successfully yielding lightweight and compact model architectures with comparable or even superior accuracy. Three categorical tasks are conducted to assess the performance of the model in distinguishing COVID-19, normal, and pneumonia CXR images: COVID-19 for COVID-19 patients, normal for perfectly healthy individuals, and pneumonia for ordinary pneumonia cases without COVID-19.

**Table 1:** The whole framework of Dense MobileNetV3.

Input	Operation	exp size	#out	SE	DB	TL	NL	stride
224^2^ × 3	conv2d	-	16	-	-	-	HS	2
112^2^ × 16	3 × 3, bneck	16	16	-	-	-	ReLU	1
112^2^ × 16	3 × 3, bneck	64	24	-	-	-	ReLU	2
56^2^ × 24	3 × 3, bneck	72	24	-	-	-	ReLU	1
56^2^ × 24	5 × 5, bneck	72	40	✓	-	-	ReLU	2
28^2^ × 40	5 × 5, bneck	120	40	✓	-	-	ReLU	1
28^2^ × 40	5 × 5, bneck	120	40	✓	-	-	ReLU	1
28^2^ × 40	3 × 3, bneck	240	80	-	-	-	HS	2
14^2^ × 80	3 × 3, bneck	200	80	-	-	-	HS	1
14^2^ × 80	3 × 3, bneck	184	80	-	-	-	HS	1
14^2^ × 80	3 × 3, bneck	184	80	-	-	-	HS	1
14^2^ × 80	3 × 3, bneck	480	112	✓	•	•	HS	1
14^2^ × 112	3 × 3, bneck	672	112	✓	•	•	HS	1
14^2^ × 112	5 × 5, bneck	672	160	✓	•	•	HS	2
7^2^ × 160	5 × 5, bneck	960	160	✓	•	•	HS	1
7^2^ × 160	5 × 5, bneck	960	160	✓	•	•	HS	1
7^2^ × 160	1 × 1, conv2d	-	960	-	-	-	HS	1
7^2^ × 960	7 × 7, pool	-	-	-	-	-	-	1
1^2^ × 960	1 × 1, conv2d, NBN	-	1280	-	-	-	HS	1
1^2^ × 1280	1 × 2, conv2d, NBN	-	3	-	-	-	-	1

Abbreviations: SE, squeeze-and-excite; DB, dense block; TL: transition layer; NL, nonlinearity; HS, h-swish; NBN, no batch normalization; s, stride. ✓ means there is this module at this location. • means the newly added module.

### 3.1 MobileNet

**Fig 1:**
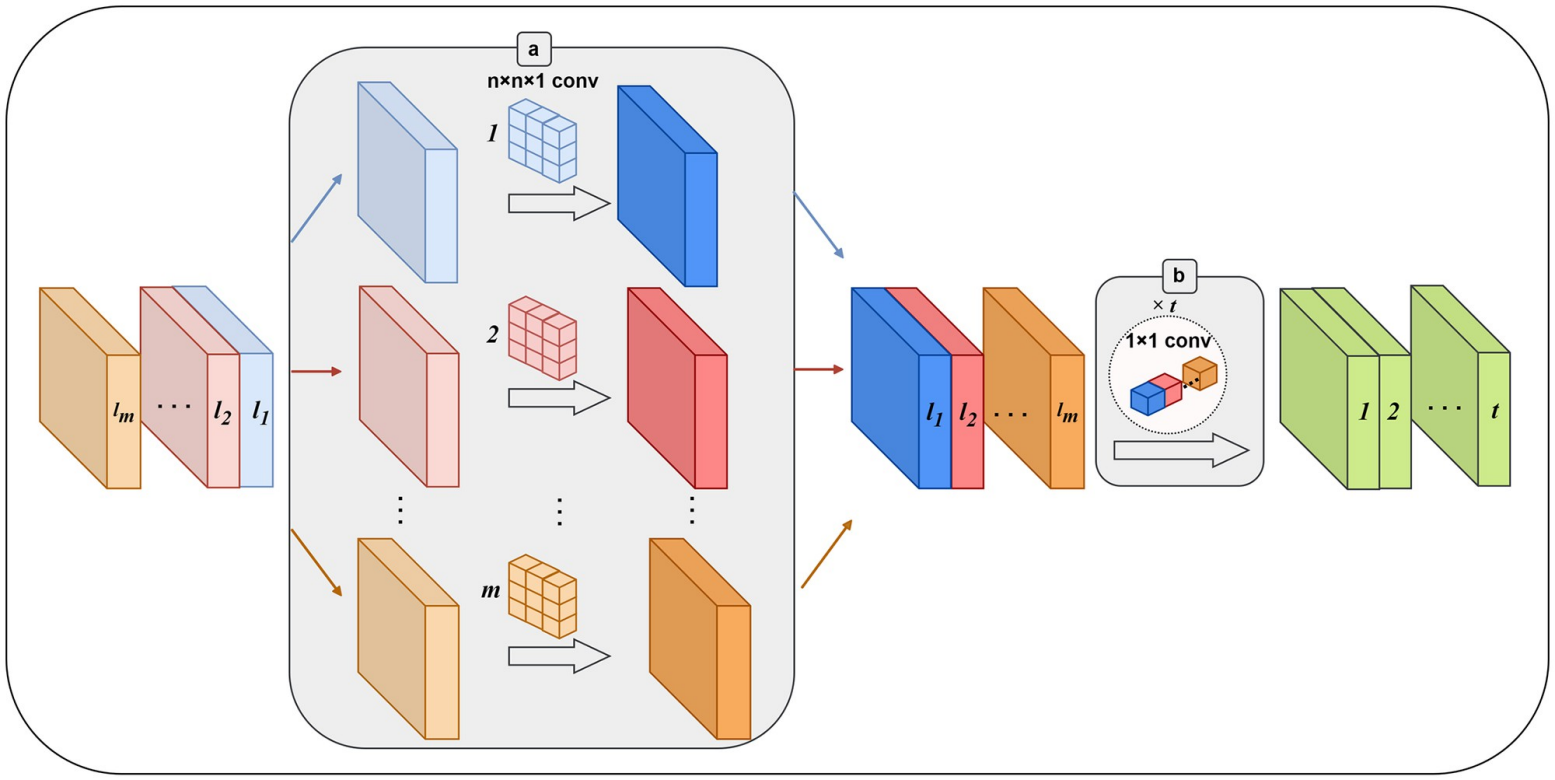
Depthwise separable convolution. (a) Depthwise convolution. (b) Pointwise convolution.

MobileNet [22] is a convolutional neural network architecture specifically suggested to address the challenges of model size and computational burden. It offers a lightweight solution that is suitable for resource-constrained devices while still achieving relatively high accuracy levels. The core structure of MobileNet is the deep separable convolution, which effectively reduces the parameters of the network. Fig 1 illustrates the process of depthwise separable convolution, consisting of two units: the depthwise convolution (2a) and the 1x1 pointwise convolution (2b). The depthwise convolution uses a separate convolutional filter on each input channel to implement the spatial filtering function. This operation captures spatial information independently for every channel. Subsequently, the pointwise convolution employs a 1x1 convolution to integrate and mix the filtered channels. By utilizing this depthwise separable convolution structure, MobileNet achieves a balance between the feature extraction capability and the parameter reduction. It enables the network to effectively capture important features while greatly decreasing the model parameter count. This reduction in parameters is crucial for efficient computation on devices with limited resources.

**Fig 2:**
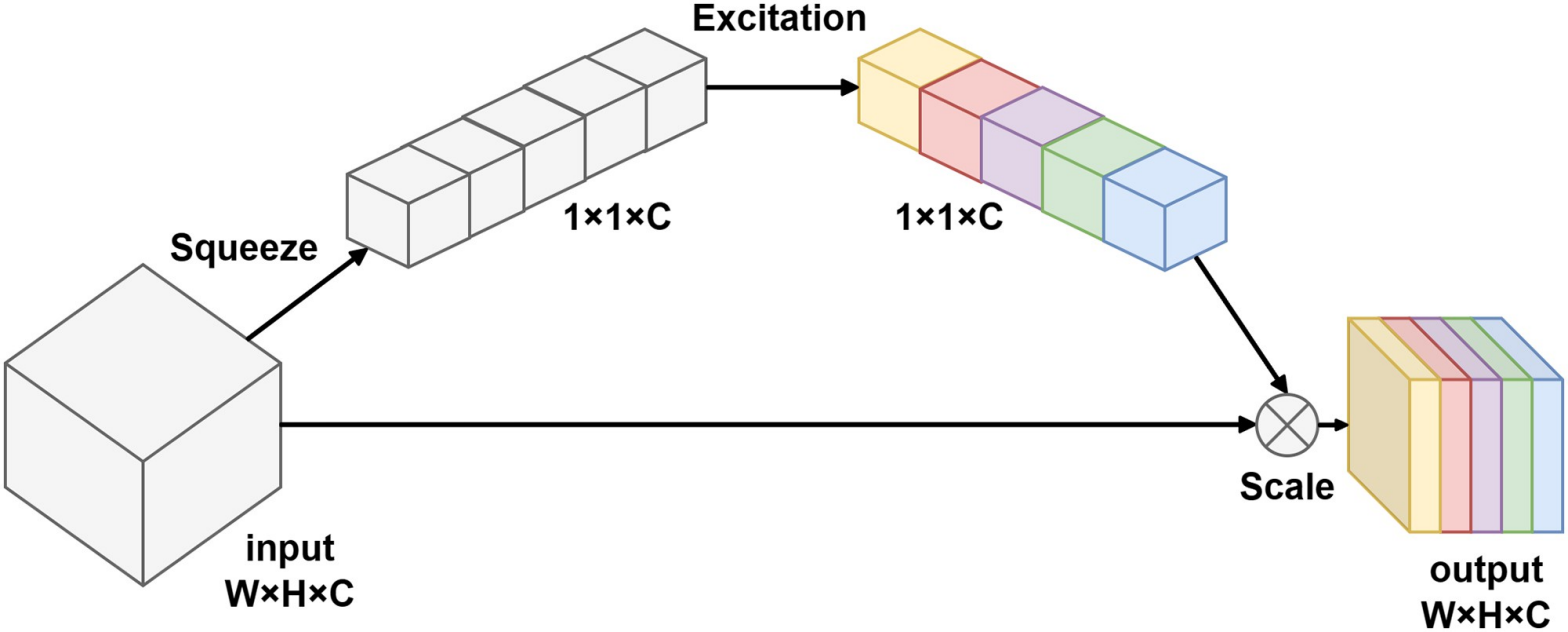
Squeeze and Excitation (SE) module. After excitation, different colors mean channels get different weights.

MobileNetV2 introduces the inverted residual block and the linear bottleneck to reduce the computational effort. The bottleneck layer of the network utilizes extended 1x1 convolutions to decrease the dimensionality of feature maps. The inverted residual block enhances the nonlinear transformation capability of the model and improves its representation ability. This allows for more effective feature extraction and representation within the network [23]. MobileNetV3 [24] incorporates the Squeeze and Excitation (SE) module, as shown in Fig 2. The SE module is based on channel feature attention and allows for the adaptive selection of significant characteristics by modifying the weights of diverse feature maps in the channel dimension. This mechanism enhances the network’s capacity to focus on important features and improves its performance. MobileNetV3 offers two models of different complexity architectures: MobileNetV3-Small and MobileNetV3-Large. The latter achieves higher accuracy on classification tasks and reduces latency compared to MobileNetV2. Therefore, in this study, the MobileNetV3-Large architecture is employed due to its superior classification performance.

### 3.2 Dense block

This paper utilizes the DenseNet to extract spatial characteristics of different dimensions. What is noteworthy is that the densely connected framework further interconnects characteristics of diverse dimensions, which performs better compared to traditional Convolutional Neural Networks in expressing the intricate linguistic relationships among varying illnesses [25].

Compared with shallow networks, the DenseNet has the ability to learn distinguishing and robust features to improve performance. It also addresses the issue of vanishing gradients by incorporating the feature reusability within the network. This is achieved by establishing direct connections from each layer to all following layers, which allows one to learn the spatial features. The dense block layer aims to keep a seamless information stream among network layers. Meanwhile the *a*th layer *P*_*i*_ obtains the feature maps from every previous layers as input, and subsequently passes the corresponding feature map to every following layer:

$$\begin{array}{c} P_{i}=Q_{i,R}(\left[x_{0},x_{1},\ldots ,x_{i-1}\right). \end{array}$$ (1)

And *Q*_*i*,*R*_(⋅) represent a composite of functions that includes BN layer, ReLU, pooling, convolution layer and [*x*_0_, *x*_1_, …, *x*_*i*−1_] indicates the composite feature map of layer [0, …, *i* − 1]. *R* represents the increasing rate, corresponds to the output feature maps generated by each composite function. In the Dense Block (*I*, *R*), which consists of *I* layer with a growth rate of *R*, different layers of composite functions and feature map concatenations are cascaded. Eq 1 shows that as the count of layers rises within the Dense Block, the concatenation operation leads to a growth in the input size of the following layers. To facilitate downsampling, a transition layer is introduced after every Dense Block. This transition layer typically consists of a batch normalization layer, a 1 × 1 convolutional layer, and a 2 × 2 average pooling layer. In the specific case of DenseNet121, which is used to extract complex spatial features, it comprises 4 dense blocks with transition layers incorporated for downsampling. This architecture achieves the efficient feature transfer and recycle and reduces the number of parameters and enhances computational efficiency.

**Fig 3:**
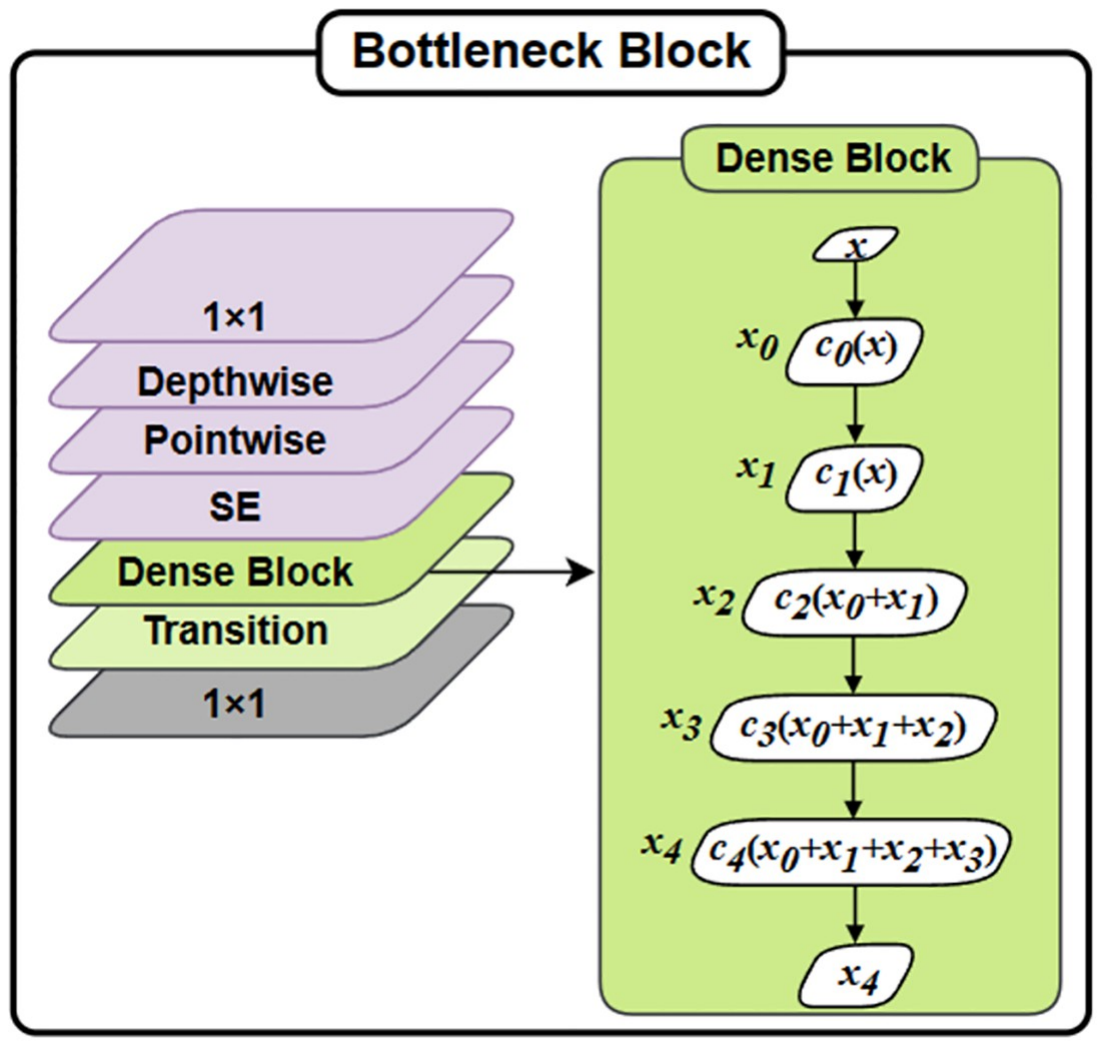
Improved bottleneck block. The dark green area on the right is the detailed process of Dense Block.

A densely connected pattern is utilized by this structure, which requires fewer parameters compared to a traditional CNN. By doing this, the network effectively reduces the need for learning unnecessary details and minimizes the number of feature maps required by each network layer. As a result, parameter efficiency is greatly enhanced. The primary advantage of these small links among layers, which located near the inputs and outputs, is to facilitate efficient backward propagation of previous features for reevaluating of feature representations. Therefore, this network structure enables the extraction of more significant characteristics. Moreover, the characteristics captured from every layer can be further melded and processed to acquire a more comprehensive descriptor. This fused descriptor can then be utilized in diverse applications to obtain improved consequences. This method establishes connections between multiple feature maps and does not incorporate explicit feature reconsideration between each layer. In contrast to integrating all feature maps, as depicted Fig 3, this research passes the output of the last layer as input to the next layer. In traditional networks, the connections are typically based on the combination of *I*(*I* + 1)/2 connections, rather than just *I* connections. Building upon the preceding layers, the feature maps of the *l*th layer can be computed, which includes [*x*_0_, *x*_1_, …, *x*_*i*−1_].

In the improved method, this study simply combines the transition layer and the dense block with the channel attention mechanism. This enables us to fully harness the benefits of the channel attention unit without significantly increasing the number of parameters. Moreover, the channel attention network is designed to be a lightweight and proficient structure, ensuring that it does not result in overfitting problems. The transition layer consists of a 1 × 1 convolutional layer and an average pooling operation with a stride of 2. This arrangement contributes to the proportional reduction in feature map size.

### 3.3 Transfer learning

In transfer learning, a model can leverage features, weights, or knowledge gained from one task to expedite training and enhance performance on another task. This approach is particularly effective for tasks with limited data or high similarity, as it maximizes the use of data from the source task. Transfer learning is widely applied in computer vision and deep learning. This approach is primarily suitable for tasks that lack sufficient samples to train from scratch, especially for the classification of medical images for uncommon or emerging diseases. For instance, researchers can use a pre-trained image classification model on the ImageNet dataset to initiate the tasks with minimal data, such as COVID-19 detection. Through transfer learning, the model benefits from well-established initial weights, resulting in swift convergence and superior outcomes on new tasks [26].

In our case, due to the scarcity of COVID-19 images, the proposed method employs pre-trained weights from the ImageNet dataset and train the model on our datasets to achieve the target task. Furthermore, our subsequent fine-tuning aims to counteract performance decline caused by pruning. The objective of fine-tuning is to optimize model performance while minimizing model size.

### 3.4 Pruning

**Fig 4:**
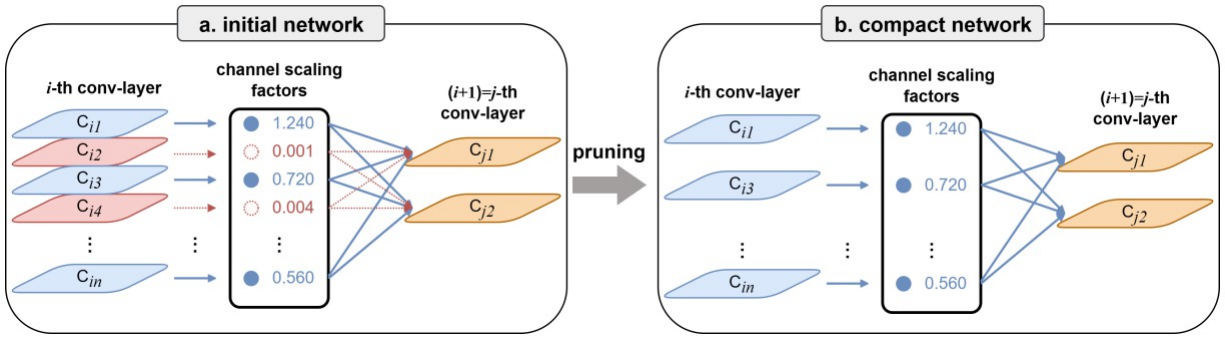
Pruning procedure. (a)Initial network. (b) Compact network. The compact network after pruning is fine-tuned to reach similar (or even better) accuracy than when trained normally.

The principal idea of the pruning algorithm is to minimize the amount of computation and parameters. Meanwhile, the performance of the network is not affected as far as possible, which can be achieved by introducing sparsity during the training stage. Sparsity refers to the existence of a large number of zero or near-zero parameters or connections in the model. Pruning involves setting some parameters or connections to zero to achieve the sparsity. The sparsity can be achieved at diverse levels, including the weight standard, kernel standard, channel standard, or layer standard. The concept of channel standard sparsity strikes an optimal equilibrium between configurability and facile deployment [27]. See Fig 4.

To attain the channel-level sparsity through pruning, it is imperative to introduce a scaling factor for each channel, which will subsequently be applied to the outcomes derived from individual channels. Consequently, this model performs pruning by trimming the input and output correlations for each channel. By adjusting these scaling factors, this model can control the importance of each channel. This design allows us to jointly optimize the network’s weights and the introduced scaling factors during the training process, thereby sparsity at the channel level.

In the actual implementation, this research performs joint training of the network’s weights and these scaling factors. This implies that our optimization objective encompasses both the predictive performance of the network and the channel sparsity. In order to promote the channel sparsity, this study applies the sparse regularization to these scaling factors. The sparse regularization effectively constrains the values of the scaling factors, causing some of them to method zero and facilitating the pruning of unimportant channels. This pruning technique enables us to obtain a more lightweight network structure and achieve higher computational efficiency during the inference phase.

Through this implementation approach, this paper can simultaneously optimize the network’s weights and the sparsity of channels during the training process, making the pruning procedure more flexible and effective. The pruned network benefits from having reduced redundant connections and parameters, leading to a more compact and efficient model. Moreover, these pruning strategies maintain the predictive performance of the network on tasks, ensuring the availability and accuracy of the pruned model. Overall, by introducing channel scaling factors and applying sparse regularization, this study can achieve effective optimization of pruning for deep neural networks, offering a better solution for applications deployed on resource-constrained devices.

### 3.5 Loss function

In addition, this study integrates label smoothing into the cross-entropy loss to alleviate the impact of similarity between different classes. The ReLU activation function is introduced in the final layer to compute probabilities, and the loss value is determined by employing the maximal likelihood input to the cross-entropy function. Label smoothing [28] is applied to adjust the initial cross-entropy. Using backpropagation, the prediction cross-entropy map between model outcomes and targets is calculated. As shown in Eq 2.

$$\begin{array}{c} H\left(x,q\right)=\sum_{i=1}^{I}-x_{i}log\left(q_{i}\right). \end{array}$$ (2)

The label of *x*_*i*_ is assigned a value of 1, representing the true class, while the remaining classes are assigned a value of 0. The *q* means the prediction result and *q*_*i*_ represents the value of the *i* prediction outcome. Specifically, when employing label smoothing, the loss function primarily focuses on the loss associated with the correct label position. This approach disregards the losses related to incorrect label positions, leading the model to excessively prioritize improving the possibility of correctly forecasting the label rather than minimizing the likelihood of falsely predicting the label. In this training process, this paper has incorporated label smoothing to account for both the losses associated with incorrect and correct label positions. This enables a more comprehensive assessment of the losses in both scenarios, for instance:

$$\begin{array}{c} x^{\prime }=\left(1-\omega \right)x+\omega d\left(I\right). \end{array}$$ (3)

In Eq 3, the variable *x*′, denotes the modified sample obtained through the process of label smoothing. For class *I*, the values of *d*(*I*) are drawn from a uniform distribution, where *ω* represents the smoothing factor. Consequently, the cross-entropy loss allows for simultaneous consideration of both the loss for the correct class and the losses associated with the other classes.

Furthermore, to minimize the potential impact of data imbalance on model performance, during the training phase, this study employs the class weight [29] technique to address the imbalance in the training data, as shown in Table 2. This technique employs higher weights for the minority classes to compensate for their relatively insufficient representation in the training data. Consequently, the loss computation turns into a weighted mean, where every instance is assigned a weight matching its respective class. Eq 4 was utilized to calculate the weight of each class.

$$\begin{array}{c} w_{i}=\frac{S}{c\times x_{i}}. \end{array}$$ (4)

where *w*_*i*_ means the weight of category *i*, *S* means the sum of training instances, *c* means the count of categories, and *x*_*i*_ means the count of instances.

**Table 2:** Specific division of the database.

Category	Training	Validation	Testing	Sum
COVID-19	2315	578	723	3616
Normal	6524	1630	2038	10192
Pneumonia	861	215	269	1345
Sum	9700	2423	3030	-

## 4 Experiments on COVID-19 detection dataset

### 4.1 Data description

**Fig 5:**
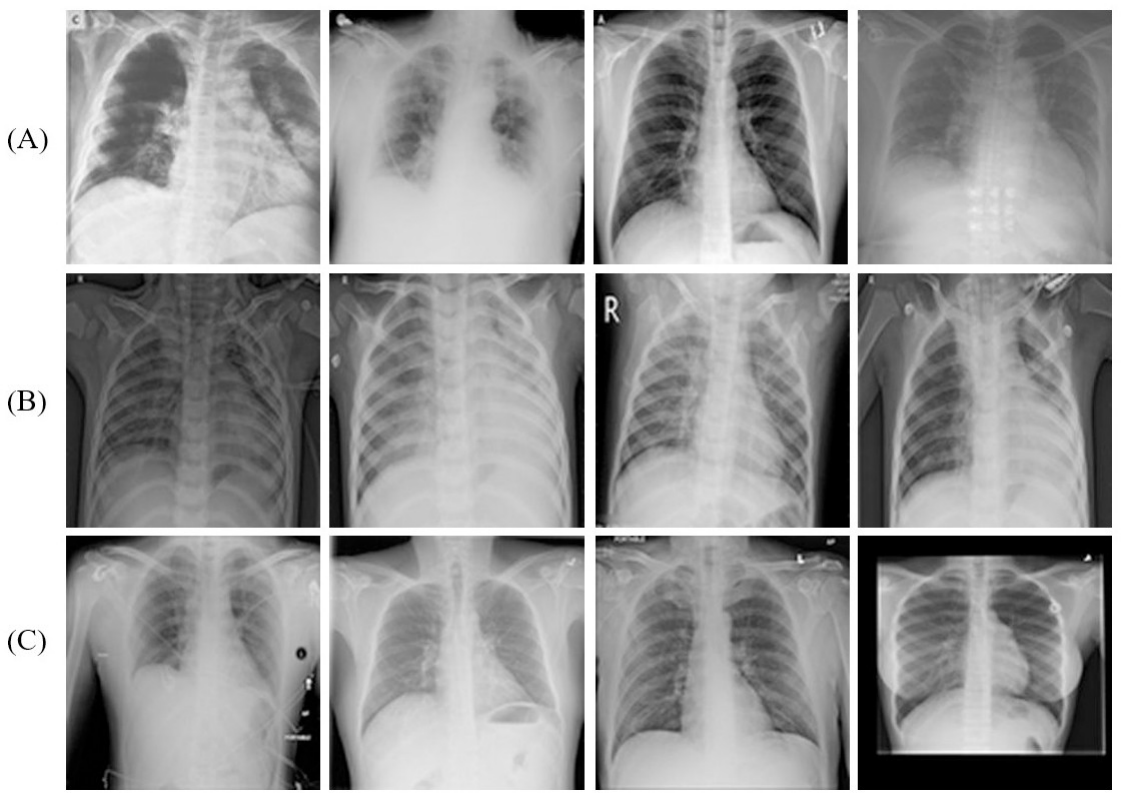
Some cases of CXR from the COVID-19 Radiography Database. (A) COVID-19 sample, (B) Pneumonia sample, and (C) Normal sample.

To conduct the experiments, this study utilizes an overt and available dataset, called COVID-19 Radiography Database. (https://www.kaggle.com/datasets/tawsifurrahman/covid19-radiography-database/discussion/223744). The development of this dataset is supervised by medical professionals, with the aim of providing a comprehensive and reliable resource for research and exploration in this domain. This dataset comprises CXR images of subjects classified into three classes: COVID-19, Viral Pneumonia, and Normal. In this study, these classes consisted of 3,616, 1,345, and 10,192 instances, respectively. The X-ray images are captured from various views and positions, as depicted in Fig 5. Therefore this paper needs to scale to a standardized size of 224 × 224 pixels so that they can be used in the MobileNetV3 channel and subsequently split into three different sets: training, validation, and testing, as illustrated in Table 2. The partition approach involved initially dividing the dataset into training and testing sets with an 80% to 20% ratio, respectively. Following this, the training dataset is further separated into training and validation sets employing the equal aforementioned proportion.

### 4.2 Training strategy

In order to evaluate the improved model, this paper begins by normalizing the images and subsequently divides them into three parts: training, validation, and testing, as mentioned previously. Then, the experiment trains the presented model using the training set and determines the optimal hyper-parameters while also making use of the validation set simultaneously. Ultimately, the researchers assess the capability of the improved model on the testing set employing a range of performance metrics explained in the subsequent section. Such as accuracy and loss with epoch, confusion matrix, and so on.

The specific network parameters are set as follows: this paper adjusts the size of all chest X-ray images to 224 × 224 × 3 as input. This method employs transfer learning to train the model, aiming to expedite the training process and achieve faster convergence of the model. This experiment sets the batch size to 32, and after every epoch, the accuracy is computed for validation. In addition, the model adopts the class weight approach to address the matter of data imbalance in the training set to reduce the possible impact on model performance. According to the results of extensive experiments conducted in this study, the final epoch is set to 80. This study utilizes the Adam optimizer and sets the minimum learning rate to 0.001. An early stop strategy [30] is used to avoid overfitting. When it identifies that there is no variation in the validation loss value, the technology stops the training process, reducing the possibility of the model overfitting. All hyper-parameters settings employed during the training process are show in Table 3.

**Table 3:** Hyper-parameter used during training.

Parameter	Value
Learn rate	0.001
Optimizer	Adam
Batch size	32
Epoches	80
Class_weight	{0:1.3967, 1:0.4956, 2:3.7557}

0: means the COVID-19 case; 1: means the normal case; 2: means the pneumonia case.

## 5 Results and discussion

In this chapter, this paper conducts a thorough assessment of the improved lightweight model Dense MobileNetV3 for COVID-19 diagnosis, utilizing broadly accepted performance metrics. The training and evaluation are conducted using the publicly available COVID-19 Radiography Database. The outcomes of multiple experiments are elaborated upon below.

### 5.1 Accuracy and loss with epoch

**Fig 6:**
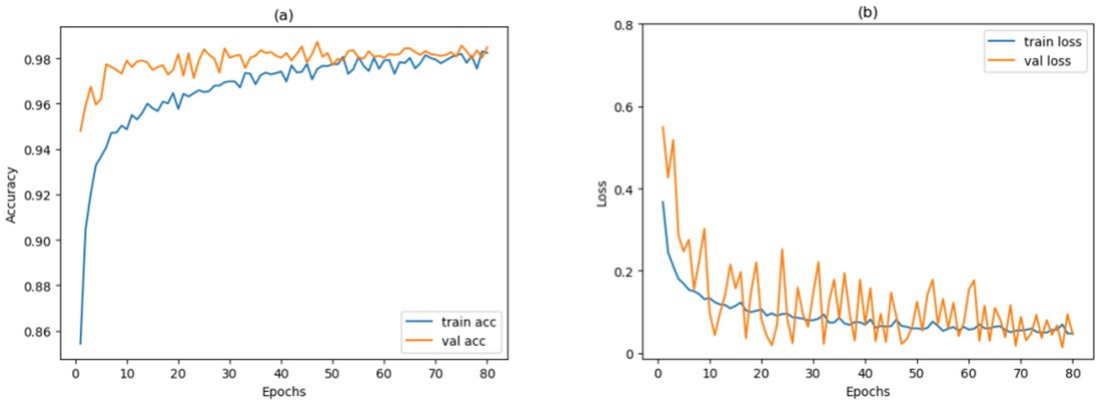
The fluctuation of accuracy and loss over epochs during training and validation.

In the training phase of a classification method, it is common to monitor and track accuracy and loss metrics over consecutive epochs to assess overfitting and observe the progress of forecasts. In this experiments, we observed that as the model’s loss value decreases during the training process, it indicates a better fit to the training data. This suggests that the model can more accurately capture patterns and relationships within the training data. Simultaneously, the high accuracy values indicating superior performance of the model on test data and its ability to generalize to unseen samples. The outcomes of these indicators can be observed in Fig 6. The tendencies depicted in the figure demonstrate that with the count of epochs growing, the accuracy and loss of the validation set at first exhibit noticeable undulations and slowly rise, resulting in higher accuracy and lower loss with minor undulations. Moreover, the tendencies reveal that the validation accuracy tends to closely align with the training accuracy across many epochs, suggesting that this method does not exhibit significant indications of underfitting or overfitting.

### 5.2 Confusion matrix evaluation

**Fig 7:**
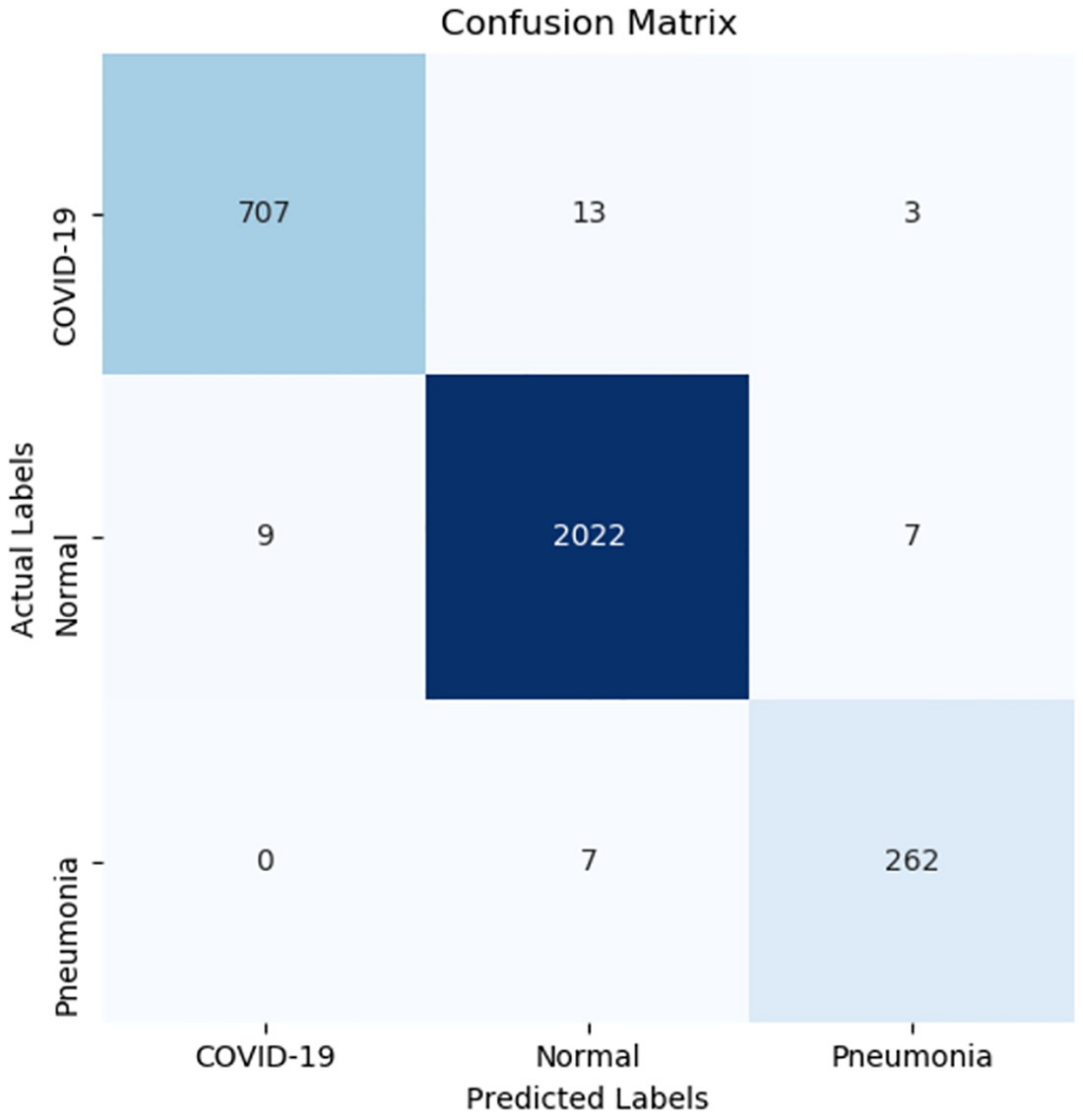
Confusion matrices distribution.

The confusion matrix is a concise representation of the prediction outcomes generated by a classifier approach, offering insights into areas where the model tends to make mistakes, i.e., mislabeling certain samples and incorrect class labels. As depicted in Fig 7, which is the result of the confusion matrices acquired from the improved model of the 3-class categorization, the model misclassified 13 COVID-19 cases from the testing sets, assigning them to the Normal, but Pneumonia is only 3. The mistake rates of these COVID-19 cases are 1.79% and 0.41%. It is obvious that the mislabeling of samples in the Normal and COVID-19 classes is more common compared with the Pneumonia category. For example, it is evident that only 0% and 2.60% of pneumonia cases were mislabeled as COVID-19 and Normal, respectively. However, despite these difficulties, the entirety performance is still good. Such as, it is evident that just 0% and 2.60% of Pneumonia cases were mislabeled as COVID-19 and Normal, severally. Similarly, 0.34% and 0.44% of Normal cases were labeled falsely as Pneumonia and COVID-19, severally. These outcomes indicate that the improved 3-class approach exhibits a relatively low error rate in every mentioned class, highlighting its impressive capability to acquire distinctive features. This is mainly because it can capture spatial features of varying scales, thereby aiding in the differentiation of highly similar features.

### 5.3 Ablation experiments

For this subsection, this study implements ablation experiments on the COVID-19 Radiography Database to assess the performance of the main elements. This article focuses on the four indicators including the accuracy, sensitivity, specificity, and precision for the COVID-19 positive category in this database. For analyzing the contributions of the improved Dense MobileNetV3 approach, Table 4 presents a quantitative comparison between the baseline model performance and the improved method. The first three rows of the table show the results of the Dense MobileNetV3 model under different conditions: without both dense block and label smoothing, without the dense block only, and label smoothing only, separately. The fourth row presents the results of our improved method, highlighting the effectiveness of the combined functionality of these methods. Notably, the network achieves the least favorable results when both dense block and label smoothing are absent. Conversely, when only the dense block is excluded, the network still produces competitive results. Additionally, the inclusion of dense block improves the accuracy of the improved Dense MobileNetV3 to 1.22%. The primary reason for this enhancement is the dense block, effectively extracting spatial features at different levels and providing a more comprehensive and accurate representation of the structures within the image. This allows for better description and recognition of the image’s structural elements.

**Table 4:** Ablation study metrics.

	Accuracy(%)	Sensitivity(%)	Specificity(%)	Precision(%)
Without Dense Block and Label Smoothing	94.07	92.23	93.68	92.25
Without Dense Block	95.64	91.94	98.18	92.62
Without Label Smoothing	96.86	94.74	98.77	95.07
**Dense MobileNetV3**	**98.71**	**97.78**	**99.60**	**98.74**

It is evident that the individual modules play a crucial role in boosting the overall performance. As seen in Table 4, the improved Dense MobileNetV3 outperforms the other ablation models, indicating that both components contribute to the enhancement and effectively work well together within the network structure. The strong feature extraction capability of our improved network is further augmented by the incorporation of label smoothing, resulting in an additional 1.85% increase in classification accuracy.

### 5.4 Pruned results analysis

As shown in Table 5, adding the dense block significantly improves the accuracy. However, it also leads to an increase in parameter count. To address this problem, the approach employs the pruning algorithm to reduce the parameter count and minimize memory consumption. This paper explores various pruning algorithms, among which weight pruning [31] can be performed offline after training, without increasing training time. However, the sparsity achieved through weight pruning is not as pronounced as with other methods, and it requires the use of dedicated sparse matrix formats during storage, thereby increasing processing costs. Neuron pruning [32], achieved by eliminating redundant neurons, contributes to a reduction in model size. However, it demands more intricate pruning strategies; otherwise, there is a risk of losing crucial information. Filter pruning [33] involves a relatively complex training and pruning process, requiring careful design of pruning strategies to potentially avoid performance degradation. Group-wise pruning [34], effective in reducing model size in some network architectures, entails a relatively complex pruning process and is not universally applicable to all network structures. Channel pruning imposes L1 regularization on the scaling factors of batch normalization layers, making it easy to implement without any changes to the network structure. Based on a comprehensive analysis of experimental results, Table 5 channel pruning demonstrates the highest accuracy performance after optimization and features a relatively simple processing flow, which is just in line perfectly with the requirements of our approach. Consequently, we have decided to adopt this method. In this paper, channel sparsity is explicitly incorporated into the optimization objective during the training process, rendering the channel pruning process smoother and minimizing accuracy loss.

**Table 5:** Comparison of the effect after using various pruning methods.

Pruning Methods	Parameters (Million)	Accuracy (%)
Weights	6.82	98.66
Neuron	5.79	97.85
Filter	6.15	98.54
Group-wise	6.37	98.12
Channel	5.94	98.71

After the channel pruning method was determined, several pruning ratios were tested to achieve the best results. This method determines that setting the channel pruning ratio to 40%. The detailed results are shown in Table 6. Through pruning, the model parameters are significantly reduced from 9.6 million to 5.9 million, successfully reducing the storage space and computational complexity, thus achieving a more lightweight model. Remarkably, despite the reduction in parameters, the model’s accuracy experienced a marginal improvement from 98.62% to 98.71%. This indicates that pruning does not significantly impact the performance of the model negatively and even has a positive effect to some extent.

**Table 6:** Comparison of parameters before and after pruning.

Model	Accuracy	Parameter(Million)	Pruned
MobileNetV3-Large	94.07	4205875	-
Dense MobileNetV3	98.62	9671819	-
40%Pruned	98.71	5948169	38.5%

### 5.5 Robust analysis

To further validate the performance and robustness of our proposed model, this model conducted a five-fold cross-validation experiment on an additional dataset. (https://www.kaggle.com/datasets/subhankarsen/novel-covid19-chestxray-repository?rvi=1) This supplementary experiment aimed to assess the performance of model in diverse data contexts. In the cross-validation experiment, this study randomly selected 500 images from each class of the dataset, creating five mutually exclusive subsets. In each iteration, this experiment used four subsets for training and reserved one subset for testing, as shown in Table 7 for specific data distribution.

**Table 7:** Dataset of five-fold cross-validation.

Class	Total number	Select Number in this paper	train	test
COVID-19	752	500	400	100
Normal	1639	500	400	100
Pneumonia	1584	500	400	100
Total	3975	1500	1200	300

This process was repeated five times to ensure that each subset was utilized as a testing set exactly once. This research computed accuracy and F1 scores for each subset to evaluate the performance of method on this distinct dataset. As depicted in Table 8, the results demonstrate that our model excelled on this additional dataset, further confirming its robustness and generalizability. These findings underscore the applicability of our approach in diverse data contexts, instilling greater confidence in its practical deployment.

**Table 8:** Results of five-fold cross-validation.

Data	Accuracy(%)	F1-score(%)
Fold 1	96.09	96.15
Fold 2	97.93	97.67
Fold 3	95.65	95.58
Fold 4	96.37	96.15
Fold 5	95.22	95.29

### 5.6 Contrasting against the previous methods

This section shows a comparative evaluation between the improved model and recently published COVID-19 detection models in the literature to assess the performance of our model. The comparative results are presented in Table 9. Due to the different data sets used by each method, it is unfair to directly compare the performance of these models. As time went on and more data samples became available, this problem has been common in multiple former papers. When the COVID-19 pandemic first emerged, obtaining a substantial number of data samples presented challenges. However, as time progressed, the availability of examples increased, and access to public repositories such as GitHub and Kaggle made it easier to acquire data. These developments greatly facilitated the pace of model improvement.

**Table 9:** Evaluate accuracy and parameters by comparing the relevant model.

Approach	Data size			Accuracy (%)	Parameters (Million)
	COVID-19	Normal	Pneumonia		
VGG19 [9]	224	504	714	93.48	143.67
VGG16 [28]	445	2880	5179	94.50	138.00
CoroNet [29]	284	310	657	95.00	33.97
InceptionV3 [14]	361	365	362	97.00	24.00
COVID-Net [15]	358	8066	5538	93.30	11.75
MobileNetV2 [16]	1200	1341	1345	94.50	3.53
EfficientNetB0 [17]	202	300	300	96.80	5.30
**Dense MobileNetV3**	**3616**	**10192**	**1345**	**98.71**	**5.94**

While acknowledging the variability in dataset sizes, it is obvious that the improved models demonstrate superior performance compared to most existing models in terms of accuracy, as indicated by the results shown in Table 9. The three-classification model, in particular, exhibits significant improvements in accuracy rates when compared to various models. It shows an improvement of 5.23%, 4.21%, 3.71%, 1.71%, 5.41%, 4.21%, and 1.91% when compared to VGG19 [9], VGG16 [35], CoroNet [36], Inception-V3 [14], COVID-Net [15], MobileNet-V2 [16], and EfficientNetB0 [18], respectively.

Furthermore, the authors can observe from Table 9 that the parameter counts of other models mentioned are between two and twenty-three times higher than that of this improved model. This is a remarkable achievement because the model can acquire a relatively high accuracy with fewer computing and memory resources. This is essential for cost-effective model development. While there are two models with fewer parameters than ours: the EfficientNetB0 had only 0.64 million fewer parameters, but the accuracy decreased by 1.91%. The other is that the improved MobileNetV2 has 2.41 million fewer parameters, but the accuracy loss is as high as 4.21%. Seeking to reduce the number of parameters can lead to a significant loss of precision due to the limitations of lightweight structures in representing complex features. However the proposed model in this paper leverages the superior performance of the MobileNetV3 structure and enhances its capability to capture spatial features at different levels by incorporating the dense block and transition layer. Additionally, the use of the cross-entropy loss function improves the model’s accuracy. Finally, the pruning algorithm further reduces unnecessary parameters on top of the lightweight model structure, achieving a high accuracy of 98.71% with 5.94 million parameters. This demonstrates a successful balance between precision and parameter count, effectively achieving the desired trade-off. This indicates that the improved approach achieves improved performance while keeping complexity and parameter usage at a reasonable level, which is essential for building economically efficient models. In lightweight networks, balancing performance optimization with the reduction of model parameters is crucial.

**Fig 8:**
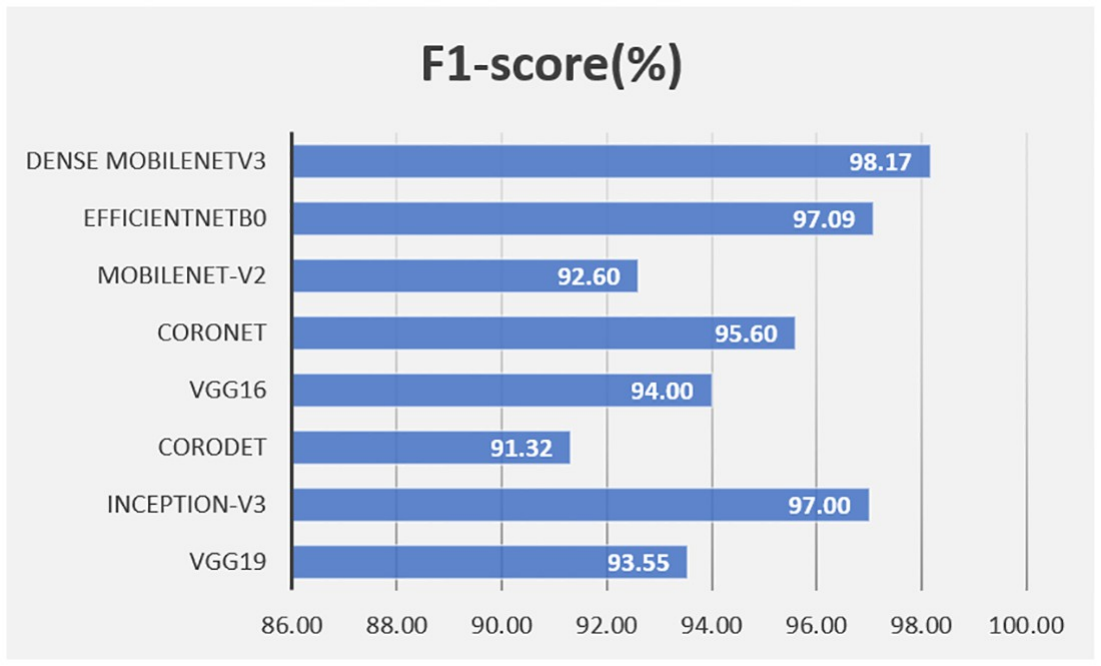
F1-score comparison.

This article further conducts a comparison with existing models by using the F1-score metric, as shown in Fig 8. The comparison reveals a significant improvement in performance for the proposed model when compared to the existing models, as clearly demonstrated in the figure.

**Fig 9:**
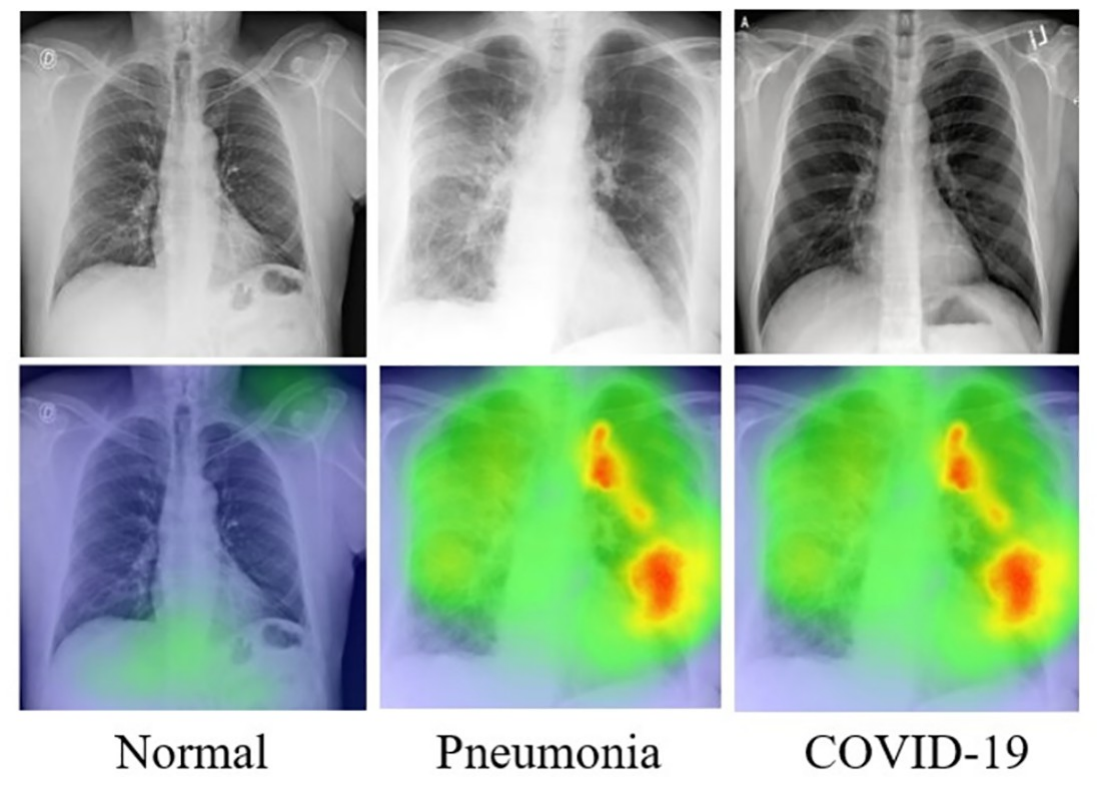
Visualization of chest X-ray images using Grad-CAM on Dense MobileNetV3 model.

In the field of medical image classification, deep learning has made significant advancements. However, its opacity and inherent lack of interpretability have long been prominent concerns. In order to enhance the interpretability of model, Selvaraju et al. [37] employed the analysis technique of Gradient-weighted Class Activation Mapping (Grad-CAM). Grad-CAM is a gradient-based interpretability tool that aids in understanding the pivotal decisions made by deep learning models in image classification. Grad-CAM highlights regions in images that are directly relevant to the model’s classification decisions, providing a visual means to explain the model’s outputs. This not only assists medical professionals in comprehending the model’s decision-making process but also enhances the model’s credibility and utility. In this study, traditional X-ray images serve as inputs, and the proposed model functions as the detection strategy. Following the label predictions by the proposed model, Grad-CAM is promptly applied to the final convolutional layer. Fig 9 illustrates the visualization of heatmaps on X-ray images using the proposed approach.

Upon analyzing this data, the researchers are prompted to consider model optimization in greater detail. Scholars should understand that reducing the number of parameters can improve computational efficiency and reduce storage overhead, which is crucial for deploying models on resource-limited devices. However, academics must also keep the trade-off between compressing parameters and maintaining model performance. In many real-world applications, model accuracy is of paramount importance, particularly in fields like medical diagnosis, in which even a minor reduction in precision can lead to severe consequences. Clearly, the proposed model achieves a better balance between high accuracy and low parameter count.

In summary, the comparisons between the improved model and the existing models demonstrate the competitiveness of the improved model. This holds particular significance for developing countries or rural areas with limited access to medical resources. In such regions, having an abundance of skilled radiologists and affordable diagnostic equipment is often a privilege. Adopting an efficient and lightweight improved model can offer valuable solutions and benefits, reducing resource needs while enhancing the accuracy and efficiency of medical services. Therefore, encouraging the adoption of the improved model in these regions is of great importance.

## 6 Conclusion

This research proposes a lightweight convolutional neural network, Dense MobileNetV3 for for efficient COVID-19 patient diagnosis using chest X-ray images. The improved model is designed as 3-class classifiers, capable of distinguishing between COVID-19, Pneumonia, and Normal individuals. Experimental results obtained on a substantial illustrate the excellent capability of the improved method. The model achieves an impressive overall accuracy rate of 98.71% across this classification task, indicating the effectiveness in accurately identifying COVID-19 cases. Some models have fewer parameters than ours, but this model obtains higher accuracy. For example, EfficientNetB0 [18] achieves an accuracy of 96.8% with 5.3 million model parameters. The parameter count is 0.64 million lower than Dense MobileNetV3, but our model exhibits a 1.91% higher accuracy compared to the reference model. We contend that the superior accuracy attained by the Dense MobileNetV3 justifies the associated computational cost. This approach achieves high accuracy while requiring a much lower count of parameters than many heavyweight models. While maintaining a low parameter count of 5.94 million, Dense MobileNetV3 achieves a high accuracy of 98.71%. This parameter count is significantly lower than that of VGG19 [9] with 143.67 million parameters, CoroNet [28] with 33.97 million parameters, and COVID-Net [15] with 11.75 million parameters. The promising results obtained in this study ·indicate the potential of the improved lightweight network for the rapid diagnosis of COVID-19. Moreover, it is well-suited for deployment on equipment with low-end configuration and power constraints, which is particularly beneficial for areas with limited medical resources and developing countries. It has the potential to facilitate early detection and prompt medical interventions, thereby contributing to efforts to control the spread of the disease.

While significant improvements have been achieved through the enhancements made to the methodology in this study, it is crucial to acknowledge certain limitations that have not been addressed. Only one dataset was used in the experiment, and its size, source, quality, and representativeness may be limited, leading to some potential biases. Furthermore, the validation of our method was limited to a specific disease and imaging modality application scenario, rendering it less universally applicable. These limitations will be the primary focus of our subsequent research endeavors.
